# Dynamic protein changes in the perihaemorrhagic zone of Surgically Treated Intracerebral Haemorrhage Patients

**DOI:** 10.1038/s41598-019-39499-2

**Published:** 2019-02-28

**Authors:** Lovisa Tobieson, Bijar Ghafouri, Peter Zsigmond, Sandro Rossitti, Jan Hillman, Niklas Marklund

**Affiliations:** 1 Department of Neurosurgery and Department of Clinical and Experimental Medicine, Linköping University, Linköping, SE-581 85 Sweden; 2 Division of Community Medicine, Department of Medical and Health Sciences, Linköping University, Pain and Rehabilitation Centre, Anaesthetics, Operations and Specialty Surgery Centre, Region Östergötland, Linköping, SE-58185 Sweden; 3 Lund University, Skåne University Hospital, Department of Clinical Sciences Lund, Neurosurgery, Lund, SE-22185 Sweden

## Abstract

The secondary injury cascades exacerbating the initial brain injury following intracerebral haemorrhage (ICH) are incompletely understood. We used dual microdialysis (MD) catheters placed in the perihaemorrhagic zone (PHZ) and in seemingly normal cortex (SNX) at time of surgical ICH evacuation in ten patients (range 26–70 years). Routine interstitial MD markers (including glucose and the lactate/pyruvate ratio) were analysed and remaining microdialysate was analysed by two-dimensional gel electrophoresis (2-DE) and nano-liquid chromatography tandem mass spectrometry (nLC-MS/MS). Two time intervals were analysed; median 2–10 hours post-surgery (time A) and median 68–76 hours post-ICH onset (time B). Using 2-DE, we quantified 232 ± 31 different protein spots. Two proteins differed between the MD catheters at time A, and 12 proteins at time B (p < 0.05). Thirteen proteins were significantly altered between time A and time B in the SNX and seven proteins in the PHZ, respectively. Using nLC-MS/MS ca 800 proteins were identified out of which 76 were present in all samples. At time A one protein was upregulated and two downregulated, and at time B, seven proteins were upregulated, and four downregulated in the PHZ compared to the SNX. Microdialysis-based proteomics is feasible for study of secondary injury mechanisms and discovery of biomarkers after ICH.

## Introduction

Current medical treatment options for intracerebral haemorrhage (ICH) include correction of coagulation disorders and, potentially, acute blood pressure lowering to prevent ICH expansion^1,2^. Although ICH removal is life-saving in selected cases, the role of surgery in improving outcome in larger cohorts of ICH patients remains controversial^3–5^. Furthermore, there are no pharmacological treatment options with proven clinical benefit available, likely since most mechanisms contributing to the ICH pathophysiology are unknown. It is plausible that the region surrounding an ICH, the perihaemorrhagic zone (PHZ), is particularly vulnerable. There is experimental and clinical evidence of an acute hypometabolic and hypoperfusion state^6–8^ including signs of mitochondrial dysfunction and metabolic failure in the PHZ^9,10^, which is also characterized by oedema, necrosis and apoptosis as well as infiltration of inflammatory cells^11^. In addition, a cascade of secondary injury factors is initiated by products of the coagulation cascade and ICH breakdown products, in particular thrombin, which results in microglia activation within hours^11^. These activated cells in turn release factors that induce breakdown of the blood-brain barrier, exacerbate the vasogenic oedema formation and induce apoptosis in both neurons and glia^11^. To develop novel treatments and to enable monitoring of ICH-related events, improved knowledge of these secondary cascades occurring in the PHZ is necessary.

Cerebral microdialysis (MD) has been extensively used in human traumatic brain injury although rarely in clinical ICH research^10,12–15^. In recent years, and with the introduction of catheters with a larger pore size, microdialysis has enabled sampling of larger peptides and proteins from the extracellular environment^16^. By studying dynamic protein changes a better understanding of the pathophysiological mechanisms is possible, as well as the exploration and characterization of biomarkers of evolving brain injury.

The proteins present in a tissue reflect which genes are expressed, and what signalling pathways are activated thus by evaluating proteins, instead of genes or mRNA, the functional expression of cells or tissues can be examined. The use of separation techniques such as 2-D gel electrophoresis allows for the differentiation and quantification of different protein isoforms. In this study, we sampled extracellular fluid using paired microdialysis catheters, one in the PHZ and one in seemingly normal and non-eloquent cortex (SNX), to investigate changes in metabolic, inflammatory and cellular pathways in the PHZ in the acute phase after surgical evacuation of ICH. Following analysis of routine low-molecular weight metabolites, the remaining microdialysate was investigated by proteomics using both a 2D-gel based and a nano-liquid chromatography tandem mass spectrometry (nLC-MS/MS) based approach to screen for a large number of proteins. We hypothesized that a number of proteins should up- or downregulate between the two MD catheter locations and over time, indicating what pathophysiological mechanisms are occurring and how they develop over time in the vulnerable PHZ surrounding a surgically evacuated ICH.

## Materials and Methods

### Ethics

The regional ethical committee in Linköping, Sweden approved the study protocol (decision number 2014/236-31). The study was carried out in accordance with relevant guidelines and regulations, including the WMA Declaration of Helsinki. Since the included ICH patients could not themselves consent to the study, a written informed consent was obtained from the patient’s closest relative.

### Patients

Patients prospectively recruited to this study were adults (>18 years) requiring emergent surgery for spontaneous intracerebral haemorrhage (ICH) using an open craniotomy. Patients with severe coagulation disorder and with a known source of bleeding such as an aneurysm or arteriovenous malformation were excluded. At the time of surgery one microdialysis catheter (10 mm CMA-71 Brain Catheter, M-Dialysis, Solna, Sweden) was inserted in the perihaemorhagic zone (PHZ) via the craniotomy, and one catheter was inserted ipsilateral to the haematoma but in seemingly normal and non-eloquent cortex, at a 45 degree angle aiming for the microdialysis membrane to cover the cortex-white matter junction, either through the same craniotomy (n = 8) or via a separate burr hole (n = 2). Four of the patients were included in a previous publication from our group evaluating energy metabolic disturbances using MD following ICH surgery^15^.

Patients were treated according to a standardized neurocritical care protocol to avoid secondary insults. This included intubation and mechanical ventilation if the patient was unconscious (Glasgow Coma Scale motor score (GCS-M) ≤5), aiming for normoventilation, normovolemia, and normothermia. Intracranial pressure (ICP) was maintained at <20 mmHg and cerebral perfusion pressure (CPP) >60 mmHg with the use of volume substitution and inotropic drugs such as norepinephrine or dobutamine, when needed. Patients received prophylaxis against thromboembolic events using intermittent pneumatic compression (IPC) and low molecular weight heparin (LMWH) from day 2 after surgery.

A routine craniotomy was performed and the ICH was evacuated by microneurosurgical technique. ICP-monitoring was achieved using either Neurovent-P parenchymal pressure monitoring device (Raumedic AG, Helmbrechts, Germany) or Bactiseal external ventricular drainage (EVD) catheter (DePuy Synthes, Raynham, USA). As it is essential for the interpretation of MD-sampling to know the location of the membrane^17,18^ a post-operative CT-scan was performed to verify MD catheter placement and for control of any ICH remnant.

To determine outcome according to modified Rankin Scale (mRS) patients or their closest relative were asked to complete a validated questionnaire at 3–6 months post ICH-onset^15,19,20^.

### Microdialysis

Microdialysis catheters of 10 mm length, with a molecular weight cut-off (MWCO) of 100 kDa (CMA-71, M-dialysis AB, Solna, Sweden) were used. The catheters were perfused per institutional routine with 5% human albumin in a water solution containing the excipients sodium chloride, N-acetyl-DL-tryptophan and caprylic acid (Albunorm, 50 g/l, Octapharma AB, Stockholm, Sweden), at a rate of 0.3 µL/min using the CMA 106 perfusion pump (M-Dialysis AB, Solna, Sweden)^16^. The first 2 hours of sampling were discarded according to consensus praxis^18,21^. Samples were collected every 2 hours for routine analysis of small molecular metabolites^18,21–23^.

### Analytical Methods

#### Metabolite analysis

Interstitial levels of small molecular metabolites (glucose, lactate, pyruvate, glycerol and glutamate) in the MD samples were analysed bedside using an ISCUS Flex® analyser (M Dialysis AB, Solna, Sweden). The limits of detection (LOD) were 1.0 μmol/L for glutamate, 0.1 mmol/L for glucose and lactate, 10 μmol/L for pyruvate and 0.22 mg/mL for glycerol. No sample preparation was needed. Metabolite concentrations in the MD samples were analysed by enzymatic methods using the ISCUS Flex® analyser, immediately after sample collection. The sample volume required for the different metabolites was 0.5 µL for glucose, 0.2 µL for lactate, 0.5 µL for pyruvate, 0.5 µL for glycerol, 1 µL for glutamate and 0.5 µL for urea. Following this analysis, the remaining MD sample (approximately 30 µL/vial) was frozen and stored at −20 °C, and typically within 2–8 weeks transferred to Eppendorf vials and stored at −86 °C until further analysis.

#### Protein analysis

For the protein analyses two vials (sampling time 4 h) were pooled for each analysis method and two time intervals (A and B) were analysed for each catheter. The first analysis (A) was performed at a median 2–10 hours (range 2–10 to 8–16 hours) after MD catheter placement (the first two vials were used for gel-based analysis and third and fourth vial were used for LC-MS/MS). The second analysis (B) was performed at a median 68–76 hours (range 52–62 to 70–80 hours) post ICH-onset (for details see Supplementary Table S1). This second analysis (B), defined as 68–76 hours post ICH onset, corresponded to a median 52 hours (range 24–64 hours) after MD catheter placement (Table 1).

**Table 1:** Patient characteristics.

Pat #	Age (years)	Co-morbidities	ICH volume (mL)	Location of ICH	Dist. PHZ-MD to ICH (mm)	Dist. SNX-MD to ICH (mm)	Side of ICH	GCS-M on arrival	GCS-M on depart	LOS in NCC (d)	Outcome (mRS)	Time ICH onset to surgery (h)	Time from surgery to A (h)	Time ICH onset to B (h)	Time from surgery to B (h)	Time ICH onset to start of MD sampling (h)	Total MD-sampling time (h)
1	68	VKA, AF, DM	90	BG	3	34	R	5	3	5	4	6	4	68	58	14	98
2	62	0	25	BG	5	22	L	5	4	6	4	22	4	70	44	30	68
3	70	PMI, APT, HT	150	BG	15	20	R	4	6	5	4	6	6	70	64	12	92
4	48	HT, pMI, KD	83	BG	10	43	R	6	6	3	6	8	4	52	40	16	48
5	60	AF, LD	145	T	9	27	L	6	5	9	6	22	6	72	24	54	42
6	62	HT, LD	102	F	5	15	R	5	6	3	3	33	6	70	34	42	48
7	55	HT, obe	87	BG	7	13	L	5	4	5	6	4	4	68	56	16	84
8	26	HT	64	BG	10	28	L	5	6	9	3	22	10	68	48	30	176
9	48	HT, CVL	57	BG	5	13	R	5	5	7	4	6	2	72	64	10	170
10	51	0	71.4	BG	8	30	R	5	6	7	3	6	4	70	62	12	162

Abbreviations; ICH = intracerebral haemorrhage; HT = hypertension, CVL = previous cerebrovascular lesion; AF = atrial fibrillation; VKA = vitamin-K antagonist (Warfarin) treatment; HepB = Hepatitis B; DM = diabetes mellitus; KD = kidney disease; LD = liver disease; obe = obesitas; pMI = previous myocardial infarction; BG = basal ganglia; T = temporal lobe; F = frontal lobe; Dist. = distance; SNX = seemingly normal cortex; MD = microdialysis probe PHZ = perihaemorrhagic zone; R = right; L = left; GCS-M = motor component of Glasgow Coma Scale score; NCC = neurocritical care; LOS = length of stay; h = hours; d = days; mRS = modified Rankin Scale assessed at 3 months post-surgery.

#### Gel-based proteomics (2D-PAGE)

Sample preparation: The dialysate samples contained a high amount of albumin since it was included in the perfusion fluid. To remove albumin, 40 µL of each samples was applied onto an albumin & IgG Depletion column (GE Healthcare, Uppsala, Sweden) according to the manufacturer´s recommendation. Briefly, 40 µL of the MD samples were diluted with binding buffer (20 mM sodium phosphate, 150 mM sodium chloride, pH 7.4) to a final volume of 100 µL. The spin column was equilibrated by adding 400 µL of binding buffer and centrifuged at 800 × g for 30 s. This equilibration step was repeated one more time and then the 100 µL of diluted MD samples was applied and the column incubated for 5 min without mixing at room temperature. The depleted MD sample was collected by centrifuging the spin column for 30 s at 800 × g. Then 100 µL of binding buffer was added to the column and after centrifugation for 30 s at 800 × g the eluate was collected. This step was repeated one more time. Finally a volume of approximately 300 µL of depleted MD sample was obtained. Total protein concentrations was measured using Bio Rad protein assay according to Bradford^24^. The dye reagent was diluted 1:5 with MilliQ water. Human albumin was used to generate a standard curve (6, 12, 25, 50 and 100 µg/mL). Of each standard solution or MD samples, 20 µL were mixed with 200 µL of the dye reagent. The concentrations were measured at 595 nm using Beckman Coulter DU 800 Spectrophotometer. The samples were desalted and concentrated to 35 µL sample volume using 3 kDa amicon spin-filter (Merck Millipore) according to the manufacturer’s recommendation. The concentrated samples were dried by speed vacuum concentrator and re-dissolved in 100 µL of urea buffer solution (9 M Urea, 4% CHAPS, 65 mM DTT, 0.1% Bromophenol blue, 0. 2% Pharmalyte 3–10). During the sample preparation all the samples were stored on ice to prevent protease activation.

Two dimensional gel electrophoresis: From each sample, 50 µg protein was analysed by 2-DE^25^. In the first dimension, proteins were separated by isoelectric point (pI) using IPGphore (GE Healthcare). Briefly, a volume of each sample corresponding to 50 µg of protein was diluted with rehydration solution (Urea 8 M, CHAPS 2%, DTT 0.3%, IPG buffer 0.5%, MilliQ, Orange G) to a final volume of 350 µl. The samples were added in the re-swelling cassette and the dried immobilized poly acrylamide gel (IPG) pH 3–10 was applied. The IPG strip was rehydrated by in-gel rehydration (according to the manufacturer’s instructions) for 12 h using low voltage (30 V). The proteins were then focused for up to 32 000 Vhs at a maximum voltage of 8000 V. IPGs were either used immediately for second dimensional analysis, or stored at −86 °C until analysed. Before the second dimensional separation the IPGs were equilibrated in a 15 mL solution containing 50 mM Tris–HCl buffer pH 6.8, 35% v/v glycerol, 6 M urea, 2% w/v SDS and 65 mM DTT for 15 min followed by an additional 15-min incubation using equilibration buffer with iodoacetamid instead of DTT and a trace of bromophenol blue was added. During this equilibration step all proteins were reduced and alkylated (carbamidomethylation). The second dimension separation was performed using 8–12% gradient sodium dodecyl sulphate polyacrylamide gel electrophoresis (SDS-PAGE) from GE Healthcare. The separation was carried out in a horizontal 2-DE set up on a Multiphore (GE Healthcare) running at 20–40 mA for about 5 hours. Separated proteins were visualized by silver staining as described previously^26^. Briefly, the gels were immediately after electrophoresis placed in a 500 mL fixation solution containing 50% methanol and 5% acetic acid in MilliQ water and incubated at room temperature with gentle shaking overnight. Then the gels were placed in 500 mL of 50% methanol for 5 min followed by 10 min incubation in MilliQ water. The gels were sensitized in 500 mL of 0.02% sodium thiosulphate for 1 minute, followed by 2 × 1 minutes washing with Milli-Q water. The gel was then placed in 0.1% silver nitrate solution for 20 min before excess of silver was washed away by 2 × 1 min in Milli-Q water. The protein pattern was developed in 0.04% formaldehyde in 2% sodium bicarbonate solution for 4 min. Finally, the reaction was stopped by incubation in 0.5% glycine for 5 min and washing with Milli-Q water for 2 × 20 min. The protein patterns were analysed as digitized images, using a charged coupled device (CCD) camera (VersaDoc™ Imaging system 4000 MP; Bio-Rad Laboratories) in combination with a computerized imaging 12-bit system designed for evaluation of 2-DE patterns. The amount of protein in a spot was assessed as background corrected optical density, integrated over all pixels in the spot and expressed as integrated optical density (IOD). In order to correct for differences in total silver stain intensity between different 2-DE images, the amounts of the compared protein spots were quantified as optical density for individual spot per total protein intensity of all spots in the same gel. Thereby ppm-values (parts per million) for all proteins were generated that were evaluated for differences between the catheters.

Protein identification by mass spectrometry:Protein spots of interest were excised from the gel, digested with trypsin (Promega/SDS Biosciences, Falkenberg, Sweden) as described previously^27^. Briefly, the gel piece was destained using 25 μL of 100 mM sodium thiosulphate and 25 μL of potassium ferricyanide for 2–3 min. Then gel piece was washed 6 × 5 min with Milli-Q water before addition of 50 μL of 200 mM ammonium bicarbonate and incubation for 20 min at room temperature. The gel piece was washed (3 × 5 min with Milli-Q water) and dehydrated with 100 µL of 100% acetonitrile (ACN) for 5 min or until the gel pieces were opaque white. The ACN was removed and the gel piece was dried in speed vacuum concentration system (Savant, Farmingdale, NY). 25 μL trypsin (20 mg/mL in 25 mM ammonium bicarbonate, Promega, Madison, WI, USA) was added, and to minimize autocatalytic activity, the samples were kept on ice for 30 min. The gel piece was incubated at 37 °C overnight. The supernatant was transferred to a separate tube and the peptides were further extracted by incubation in 50% ACN/5% trifluoroacetic acid (TFA, Sigma-Aldrich) for 3 h at room temperature. The supernatant from the two steps was then pooled and dried in Speed vacuum until complete dryness. The trypsinated peptides were dissolved in 6 μL of 0.1% formic acid (FA) and were applied to a nano-flow HPLC system, EASY-nLC II (Thermo Scientific) in conjugation with the mass spectrometer, LTQ Orbitrap Velos Pro hybrid mass spectrometer (Thermo Scientific) with a nano-electrospray source as described previously^28^. The peptides were separated on a C18 column (100 mm × 75 µM, particle size 5 µM). The flow rate was set to 300 nL/min and the gradient buffer contained 0.1% FA in water (buffer A) and 0.1% FA in ACN (buffer B). Buffer B was used in a linear gradient (0–100%) for 30 min to separate the peptides. Database searching was performed using MaxQuant version 1.5 with trypsin as digestion enzyme against a human taxonomy of the SwissProt database. The following parameters were used; maximum two missed cleavages; fragment ion mass tolerance 0.5 Da; parent ion mass tolerance 6 ppm; fixed modification- carbamidomethylation of cysteine; variable modifications - N-terminal acetylation and methionine oxidation. Data were filtered at 1% false discovery rate. Identifications were based on a minimum of two unique peptides.

#### Non-Gel based Proteomics (shot-gun proteomics)

Sample preparation: Dialysate samples (30 µL) were subjected to the albumin removal column as described above. After albumin depletion samples were subjected to a 3 kDa Amicon spin-filter (Merck Millipore) to desalt and concentrate the protein contents. The protein concentrations were measured using Bio Rad protein assay as described above and the desalted proteins were dried by speed vacuum concentrator, re-dissolved in 100 µL of 8 M urea in 25 mM ammonium bicarbonate and incubated at room temperature for at least 1 hour. The denaturized proteins were reduced by adding 2 µL of a stock solution of 1.25 M DTT which ended in a final concentration of 25 mM DTT for 15 minutes and alkylated with 2 µL of 3.75 M iodacetamide stock solution (final concentration 75 mM) for an additional 15 minutes. The samples were diluted 8 times with 25 mM ammonium bicarbonate and digested with trypsin (1:25, w/w trypsin/protein). The digested peptides were dried, reconstituted in 50 µL of 0.1% of formic acid in MilliQ water and approximately 0.25 µg was subjected to LC-MS/MS analysis.

LC-MS/MS analysis: Peptides were separated by reverse phase chromatography on a 20 mm × 100 µm C18 pre column followed by a 100 mm × 75 µm C18 column with particle size 5 µm (NanoSeparatoons, Nieuwkoop, Netherlands) at a flow rate 300 nL/min. EASY-nLC II (Thermo Scientific) by linear gradient of 0.1% formic acid in water (A) and 0.1% formic acid in acetonitrile (B) (0–100% B in 90 min). Automated online analyses were performed with a LTQ Orbitrap Velos Pro hybrid mass spectrometer (Thermo Scientific).

Protein identification: Raw files were searched using Sequest HT in Proteome Discoverer (Thermo Fisher Scientific, San Jose, CS, USA; version 1.4.0.288) against Human Uniprot 160707 (available at UniProtKB website: http://www.uniprot.org/taxonomy/9606) with the following parameters: semi trypsin was used as digestion enzyme; maximum number of missed cleavages 2; fragment ion mass tolerance 0.60 Da; parent ion mass tolerance 10.0 ppm; fixed modification- carbamidomethylation of cysteine; variable modifications - N-terminal acetylation. Data were filtered at 1% false discovery rate, high peptide confidence; rank 1 peptides in top scored proteins. Identified proteins were filtered using SCAFFOLD (version 1.4.0.288; Proteome Software Inc., Portland, OR, USA). Identifications were based on a minimum of 1 unique peptides, 90% peptide identification probability (using the Scaffold Local FDR algorithm), and 99% protein identification probability (using the Protein Prophet algorithm), resulting in a 0.0% decoy FDR. The label-free quantitative analysis of peptides was performed by spectral counting analysis, using normalized average total ion current (TIC) calculated for each protein to normalize run-to-run variations^29^ and quantitative differences were statistically analysed. Identified proteins were categorized according to gene ontology terms.

For analysis of protein networks, interactions and known involvement in biological processes of the identified proteins, the STRING CONSORTIUM data base was used.

### Statistical Analysis

Data was grouped according to location of catheter (perihaemorrhagic (PHZ) vs. seemingly normal cortex (SNX)) and time intervals for MD sampling (A vs. B) thus resulting in four groups; PHZ-A, PHZ-B, SNX-A, and SNX-B. Mann-Whitney U nonparametric test was used as statistical method to calculate significant differences in protein expression (gel based proteomics) between samples from different catheters. Wilcoxon signed-rank nonparametric test was used for comparison between samples from early and late time point. A p < 0.05 was considered statistically significant. Data was analysed using IBM SPSS Statistics 20 (IBM, Kista, Sweden).

Microdialysis data (glucose, lactate, pyruvate, glutamate, glycerol) were analysed using a mixed model linear (MML) approach because of the hierarchical organization of data with catheter location as fixed effect, patient number as subject level and random effect. Correlations were described using Pearson’s Correlation coefficient.

Multivariate statistical analysis of LC-based proteomic results was done using principal component analysis (PCA) and orthogonal partial least square discriminant analysis (OPLS**–**DA) with the software for omics analysis; SIMCA+ version 14 (Umetrics, Umeå, Sweden). The procedure to compute multivariate correlation models has been described earlier^30^ and is in accordance with Wheelock and Wheelock^31^. To validate the OPLS-model cross-validated analysis of variance (CV-ANOVA) was used. The model was considered of significant importance if the CV-ANOVA had p < 0.05.

## Results

**Figure 1:**
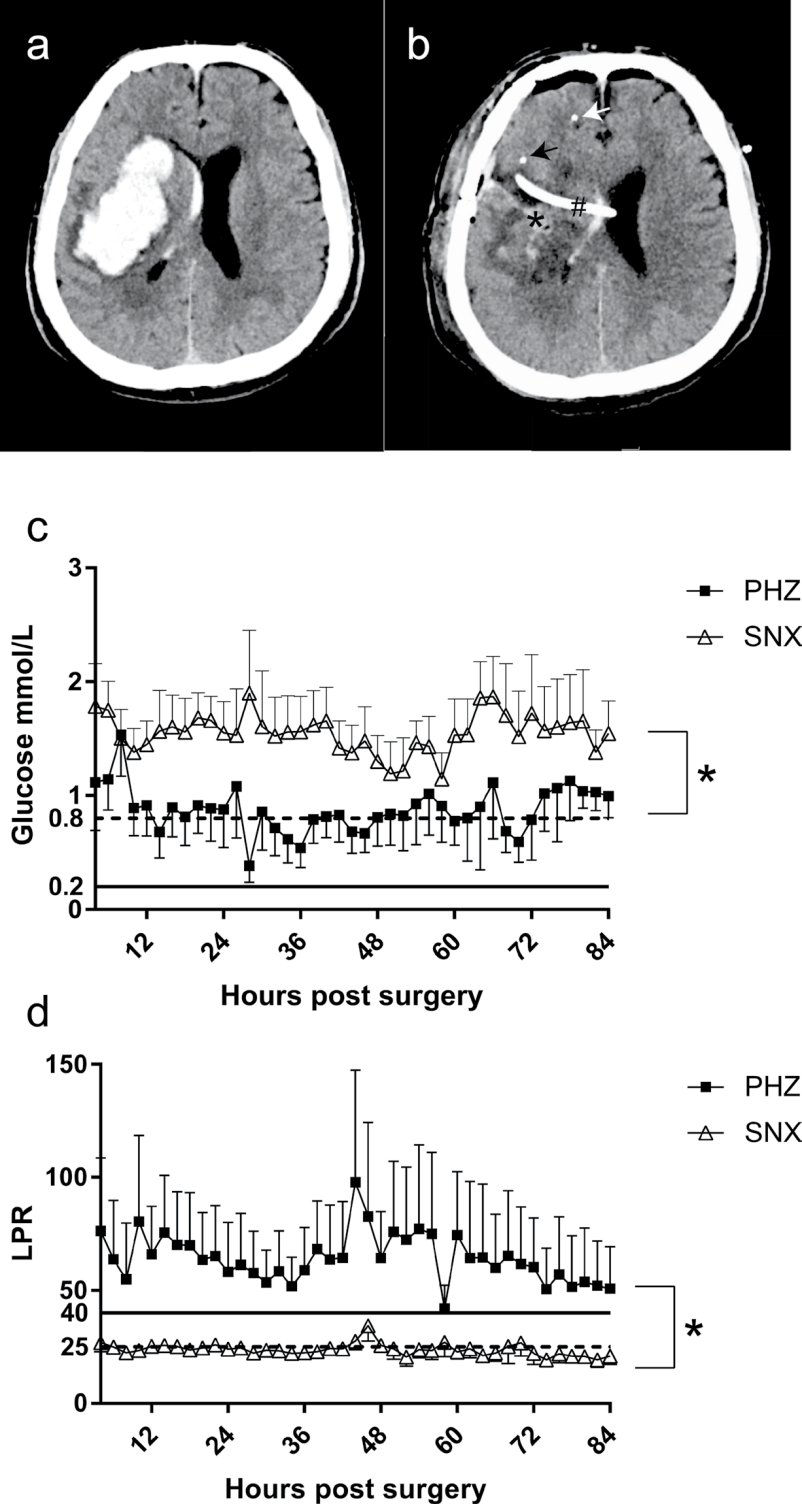
(**a**) Preoperative CT-image, (**b**) Post-operative CT-image showing EVD and MD-catheters, (**c**) MD-glucose, (**d**) MD-LPR. (**a**) An intracerebral haemorrhage in the basal ganglia in the right hemisphere of a 48-year old man with hypertension. (**b**) Post-operative CT-scan at 48 hours post-surgery. The evacuated haematoma is indicated by* and the microdialysis catheters with arrows; the catheter placed in the perihaemorrhagic zone (PHZ) in black, the catheter in the seemingly normal cortex (SNX) in white. There is an external ventricular drainage (EVD) (#) for intracranial pressure monitoring inserted through the surgical access into the ventricle at the time of surgery. Routine low-molecular weight analyses including (**c**) MD-glucose levels which are consistently lower in the PHZ compared to the SNX as expected, although consistently above the MD-glucose levels considered critical (0.2 mmol/L), indicated with a solid line. The 0.8 mmol/L indicated with a dotted line in (**c**) is considered a warning sign for low MD-Glucose. (**d**) MD- lactate/pyruvate ratio (LPR), a common indicator of energy metabolic disturbance, was consistently higher and above the critical LPR of 40 (indicated by a solid line) in the PHZ as previously shown^15^. Error bars represent standard error of the mean (S.E.M.).

Ten patients (median age 57.5; range 26–70 years; 9 males and 1 female) were included between November 2014 and July 2015. Patients and radiological characteristics are presented in Table 1. Typical pre- and post-operative computed tomography (CT) scans are shown in Fig. 1a,b, where the placement of the two MD catheters is shown. Seven patients received an ICP-monitoring device (EVD n = 2 and parenchymal ICP monitoring device n = 5). No patient had a significantly increased ICP post-operatively (data not shown).

### Low-molecular weight MD analyses

The analysis included routine assessment of low-molecular weight analyses, data presented for the first 84 h following MD insertion for glucose (Fig. 1c) and the lactate-pyruvate ratio (LPR) (Fig. 1d) in the seemingly normal cortex (SNX) and the perihaemorrhagic zone (PHZ). This data reveals an energy metabolic impairment in the PHZ with a pathologically increased LPR despite a glucose level above the critical cut-off level of 0.2 mmol/L, both in the PHZ and the SNX^21^. Urea was used to monitor the performance of the microdialysis catheter, according to established methods^32^. Only 2 vials were excluded due to deviating urea levels.

Glycerol and glutamate levels were also elevated in the PHZ compared to SNX (p < 0.05; Supplementary Fig. S2) throughout the evaluated time period.

### Proteomic analysis

#### Identified proteins in brain dialysate using 2-DE

**Figure 2:**
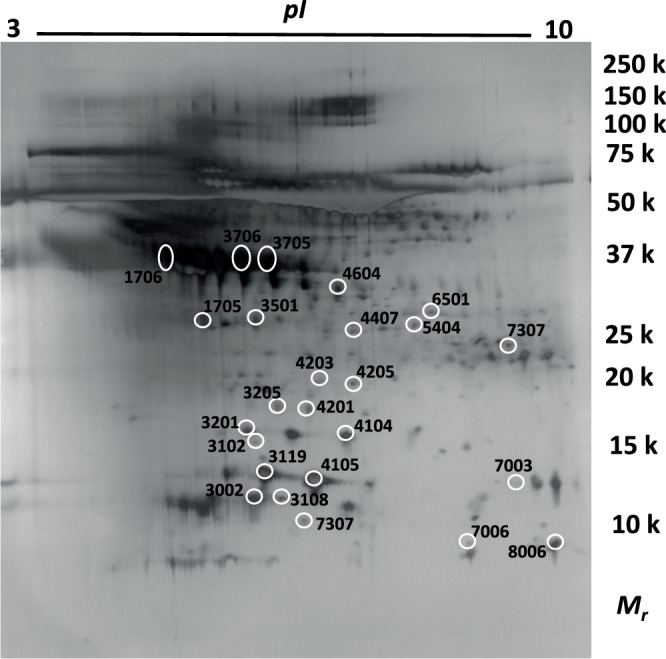
2-DE PAGE gel. A typical protein pattern for the interstitial fluid from an ICH patient. The proteins are separated by two-dimensional gel electrophoresis and stained by silver. The marked spots are significantly altered proteins and the numbers refer to the identified proteins in Table 2. The edges of the image have been slightly cropped. To view the entire original image see Supplementary Fig. S8.

The average protein concentration after albumin removal was 1.5 ± 1.2 g/L. The average protein concentration in each group was; SNX-A 1.0 ± 1.1 g/L, SNX-B 2.2 ± 1.3 g/L, PHZ-A 1.2 ± 1.1 g/L, PHZ-B 1.5 ± 1.0 g/L, The protein concentration was lower in the SNX-A when compared to SNX-B (p = 0.006) although there were no other differences among the groups. More than 200 protein spots were detected in all samples (232 ± 31). There were no significant differences in the number of spots between the four groups (PHZ-A = 231 ± 30; PHZ-B = 234 ± 36; SNX-A = 226 ± 37; SNX-B = 237 ± 23). A typical 2-DE protein map from the brain dialysate is shown in Fig. 2.

High amount of albumin (molecular weight (MW) 66 kDa) was detected in all samples despite the attempts to deplete albumin; therefore the gels were cropped and only protein spots in the area 10–50 kDa were included in the spot quantification and statistical analysis.

To investigate possible differences in the protein expression levels between the groups, protein spots present in at least 50% of gels in either group were matched, quantified for intensity, and compared between the groups. Protein spots were quantified according to the integral of the optical density over the spot area (IOD) and presented as percentage of total density in gel image.

Low molecular weight MD-analyses were investigated for association with the IOD of significantly changed proteins and only MD-LPR (r = 0.6) and MD-glutamate (r = 0.83) showed a correlation with Haemoglobin beta-subunit IOD.

#### Identified proteins in brain dialysate using LC-MS/MS

The analysis by nLC-MS/MS resulted in identification of about 800 proteins (Supplementary Dataset S3). The identified proteins clustered to 20 groups based on biological processes (Supplementary Fig. S4). In total, 76 proteins identified by LC-MS/MS were well represented in all samples and were included for the statistical comparisons between the groups.

#### Protein changes between the perihaemorrhagic zone and seemingly normal cortex

**Figure 3:**
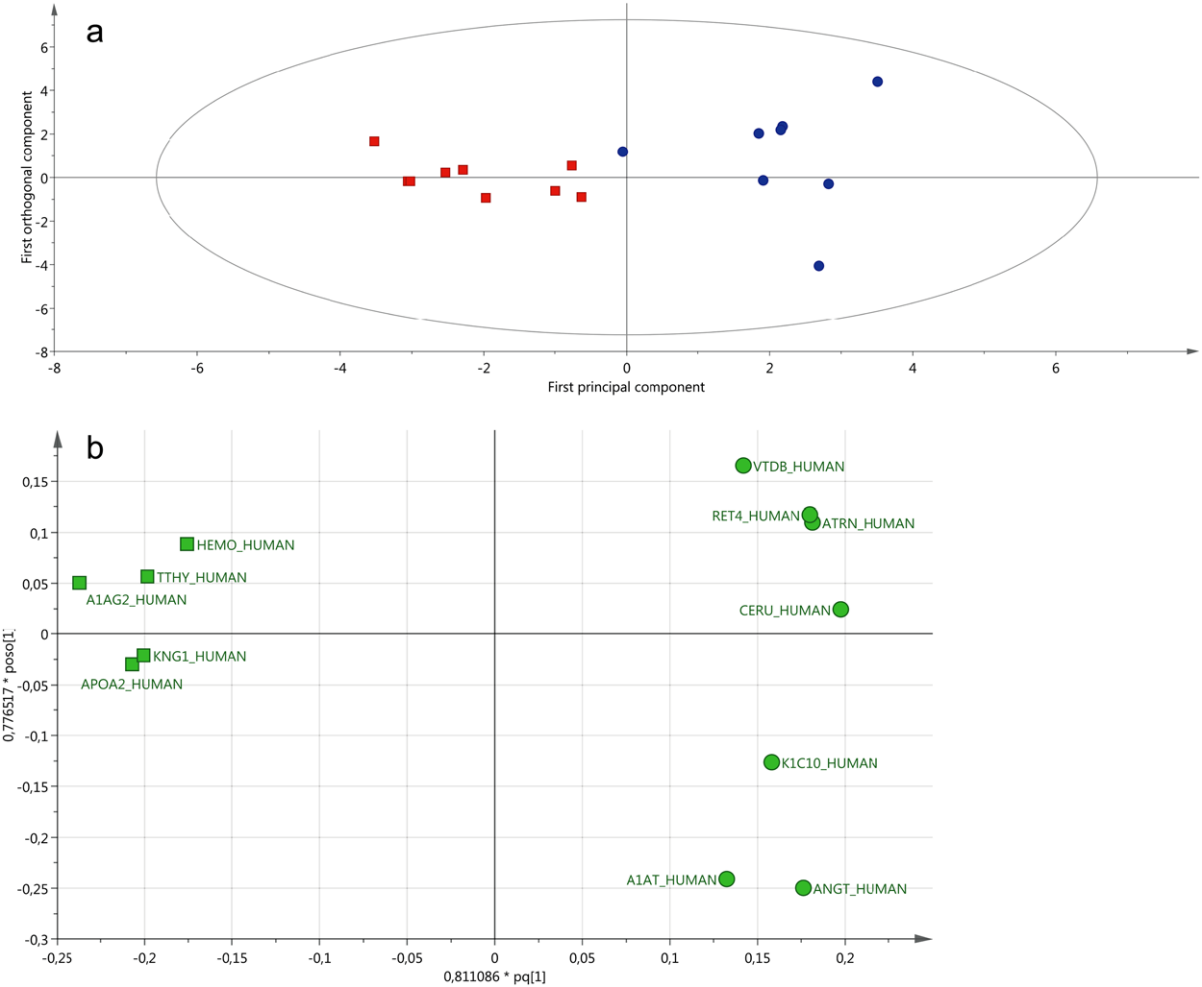
OPLS-DA of PHZ-B vs SNX-B; (**a**) Discriminant separation. (**b**) Loading plot for proteins VIP>1. (**a**) An OPLS-DA (Orthogonal Partial Least Square Discriminant Analysis; the first principal component [1] vs. the first orthogonal component to [1]) model showing the discriminant separation between PHZ-B (blue filled circles) and SNX-B (red filled squares). The longitudinal dimension (Y-axis) shows the interclass discrimination, and the latitudinal dimension (X-axis) shows the intraclass discrimination between PHZ-B and SNX-B. (**b**) Loading plot corresponding to significant proteins, with a variable of importance (VIP) value > 1. The circles correspond to the most important protein for PHZ-B and the squares indicate proteins important for SNX-B. Seven proteins; angiotensinogen (ANGT_HUMAN), ceruloplasmin (CERU_HUMAN), attractin (ATRN_HUMAN), retinol-binding protein 4 (RET4_HUMAN), alpha-1 antitrypsin (A1AT_HUMAN), ephrin type-A receptor 4 (EPHA4_HUMAN) and vitamin D-binding protein (VTDB_HUMAN) were associated with PHZ-B. Five proteins; alpha-1-acid glycoprotein 2 (A1AG2_HUMAN), apolipoprotein AII (APOA2_HUMAN), kininogen-1 (KNG1_HUMAN), transthyretin (TTHY_HUMAN) and hemopexin (HEMO_HUMAN) were associated with SNX-B dialysate samples. Abbreviations: PHZ-B = perihaemorrhagic zone at time interval 68–76 (range 50–62 to 70–80) hours post-ICH; SNX-B = seemingly normal cortex at time interval 68–76 (range 50–62 to 70–80) hours post-ICH; VIP = variable importance in projection.

Statistical analysis of the 2-DE/MS based data showed that at the early post-surgical time interval (A; median 2–10 (range 2–10 to 8–16) hours post-surgery), only two proteins were upregulated in the PHZ compared to the SNX. At the later time point (B; median 68–76 (range 50–62 to 70–80) hours post ICH-onset), two proteins were upregulated and ten proteins were downregulated in the PHZ compared to the SNX (Table 2). The mean optical density (OD) and standard deviation for these protein spots is presented in Supplementary Table S5A. Multivariate statistical analysis of the LC-MS/MS based data showed a clear separation between the samples at PHZ-B and SNX-B (Fig. 3). A total of 12 proteins (Table 3) had a variable of importance (VIP) >1 and were considered important for the group separation (Fig. 3b, Supplementary Fig. S6A,B). Those proteins together explained 82% (R^2^) of the variation with a prediction of 60% (Q^2^). The CV-ANOVA revealed that the model was highly significant (p = 0.012). Several of the proteins with highest VIP were also found to be significantly changed (p < 0.05) according to the univariate statistics (Table 4). The mean peak intensity and standard deviation for each of the proteins presented in Table 4 is shown in Supplementary Table S5B. The OPLS-DA comparison analysis between the samples from PHZ-A and SNX-A revealed no statistically significant model. The univariate analysis showed that the levels of one protein was up-regulated and two proteins were downregulated in PHZ-A compared to SNX-A (Table 4). The known or assumed biological actions and processes according to STRING of all proteins presented in Tables 2 and 4 are listed in Supplementary Table S7.

**Table 2:** Protein changes gel-based proteomics.

Spot no.	Protein name	UniProt ID	PHZ-A vs. SNX-A	PHZ-B vs. SNX-B	SNX-B vs. SNX-A	PHZ-B vs. PHZ-A
4604	Haptoglobin	P00738		↓**		
6501	Kinesin-like protein KIF12	Q96FN5		↓**	↑**	
5404	Inactive caspase-12	Q6UXS9		↓**		
3119	Transthyretin	P02766		↑**	↓**	
4105	Transthyretin	P02766		↑**		↑**
4201	Haptoglobin-related protein	P00739		↓**	↑**	
7307	Myelin protein P0	P25189-2		↓**		
3501	Protein AMBP	P02760		↓**		
4205	Ig alpha-1 chain C region	P01876		↓**	↑**	
3205	Desmoplakin	P15924		↓**		
4203	Ig alpha-1 chain C region	P01876		↓**	↑**	
4407	unidentified			↓**	↑**	
3002	Haptoglobin-related protein	J3KTC3	↑**			
7003	Haemoglobin subunit beta	P68871	↑**			
1705	Haptoglobin	P00738			↓**	↓**
3005	Ig alpha-2 chain C region (Ig like-2 domain)	P01877			↑**	
3108	Transthyretin	P02766			↑**	
3201	Haptoglobin	P00738			↑**	
4104	Haptoglobin	P00738			↑**	
7006	Dermcidin	P81605			↑**	
8006	Haemoglobin subunit beta	P68871			↑**	
1706	Haptoglobin	P00738				↓**
2703	Haptoglobin	P00738				↓**
3102	Haptoglobin	P00738				↑**
3705	Alpha-1-antitrypsin	P01009				↓**
3706	Complement C3	P01024				↓**

Significantly changed protein spot intensities using gel-based technique. The arrows indicate up- (↑) and down (↓) regulated proteins. Comparisons between location (PHZ/SNX) and time points (A/B) are illustrated. PHZ-A = Perihaemorrhagic zone at start of monitoring (median 2–10 (range 2–10 to 8–16) hours); PHZ -B = Perihaemorrhagic zone at time interval 68–76 (range 50–62 to 70–80) hours post ICH-onset; SNX-A = Seemingly normal cortex at start of monitoring (median interval 2–10 (range 2–10 to 8–16) hours); SNX-B = seemingly normal cortex at median time interval 68–76 (range 50–62 to 70–80) hours post ICH-onset. (**p < 0.05).

**Table 3:** The most important proteins (VIP > 1) for group separation (PHZ-B vs. SNX-B).

Protein name	Uniprot ID	VIP	PHZ-B *vs*. SNX-B
Alpha-1-acid glycoprotein 2	A1AG2_HUMAN	1.4605	↓
Angiotensinogen	ANGT_HUMAN	1.33639	↑*
Apolipoprotein A-II	APOA2_HUMAN	1.27218	↓*
Kininogen-1	KNG1_HUMAN	1.22996	↓*
Transthyretin	TTHY_HUMAN	1.22557	↓*
Ceruloplasmin	CERU_HUMAN	1.21031	↓
Attractin	ATRN_HUMAN	1.16447	↓
Retinol-binding protein 4	RET4_HUMAN	1.16051	↑*
Alpha-1-antitrypsin	A1AT_HUMAN	1.11074	↑*
Hemopexin	HEMO_HUMAN	1.10939	↓
Ephrin type-A receptor 4	EPHA4_HUMAN	1.06857	↑
Vitamin D-binding protein	VTDB_HUMAN	1.01255	↓

The most important proteins for group separation (PHZ-B vs. SNX-B) with VIP-value analysed by multivariate statistic. Arrows indicate upregulated (↑) or downregulated (↓) proteins. * indicates significant (p < 0.05) proteins according to univariate statistic. Abbreviations: PHZ-B = perihaemorrhagic zone at time interval 68–76 (range 50–62 to 70–80) hours post-ICH; SNX-B = seemingly normal cortex at time interval 68–76 (range 50–62 to 70–80) hours post-ICH; VIP = variable of importance in projection.

**Table 4:** Protein changes LC-MS/MS.

Protein name	Uniprot ID	PHZ-A *vs*. SNX-A	PHZ-B *vs*. SNX-B	SNX-B *vs*. SNX-A	PHZ-B *vs*. PHZ-A
Angiotensinogen	ANGT_HUMAN		↑**		↑*
Transthyretin	TTHY_HUMAN		↓**		
Protocadherin Fat 4	FAT4_HUMAN		↑**		
Transient receptor potential cation channel subfamily M member 3	TRPM3_HUMAN		↑**		
Apolipoprotein A-II	APOA2_HUMAN		↓**		
Kininogen-1	KNG1_HUMAN		↓**		
Alpha-1-antitrypsin	A1AT_HUMAN		↑**		
Bromodomain and WD repeat-containing protein 1	BRWD1_HUMAN		↓**		
Fetuin-B	FETUB_HUMAN		↑*		
Phosphatidylethanolamine-binding protein 1	PEBP1_HUMAN		↑*		
Protocadherin alpha-13	PCDAD_HUMAN		↑*		
Ig heavy chain V-III region CAM	HV307_HUMAN		↑*		
Retinol-binding protein 4	RET4_HUMAN		↑*		
Ig kappa chain C region	IGKC_HUMAN		↑*		
AN1-type zinc finger protein 4	ZFAN4_HUMAN	↑**			↓*
Beta-2-glycoprotein 1	APOH_HUMAN	↓*			
Ig heavy chain V-II region	HV201_HUMAN	↓*			
Ankyrin repeat and KH domain-containing protein 1	ANKH1_HUMAN				↑*
Mucin-5B	MUC5B_HUMAN			↓**	
Cysteine-rich secretory protein 3	CRIS3_HUMAN			↓*	
Delta-like protein 1	DLL1_HUMAN			↑*	
Protein SON	SON_HUMAN			↑*	

All statistically significantly changed proteins (*p < 0.1; **p < 0.05) using nLC-MS/MS technique. Comparisons between location (PHZ vs SNX) and post-surgery time points (A vs B) are illustrated. PHZ-A = Perihaemorrhagic zone at start of monitoring (median 2–10 (range 2–10 to 8–16) hours); PHZ-B = Perihaemorrhagic zone at 68–76 (range 50–62 to 70–80) hours post ICH-onset; SNX-A = Seemingly normal cortex at start of monitoring (2–10 (range 2–10 to 8–16) hours); SNX-B = seemingly normal cortex at 68–76 (range 50–62 to 70–80) hours post ICH-onset. Arrows indicate upregulated (↑) or downregulated (↓) proteins.

#### Protein changes over time

As shown in Table 2, the 2-DE/MS based results showed that there were 11 proteins upregulated, and two proteins downregulated, over time in the SNX. In the PHZ there were two proteins upregulated and five proteins downregulated over time when comparing time A (median 2–10 (range 2–10 to 8–16) hours post-surgery) with time B (median 68–76 (range 50–62 to 70–80) hours post ICH-onset). Univariate statistical data analysis was used to evaluate time dependent protein changes for the LC-MS/MS based proteomic analysis. As shown in Table 4 two proteins were upregulated, and two proteins downregulated, over time in seemingly normal cortex (SNX). In the perihaemorrhagic zone (PHZ) two proteins were upregulated and one protein downregulated over time when comparing time A to time B.

#### Protein isoforms

**Figure 4:**
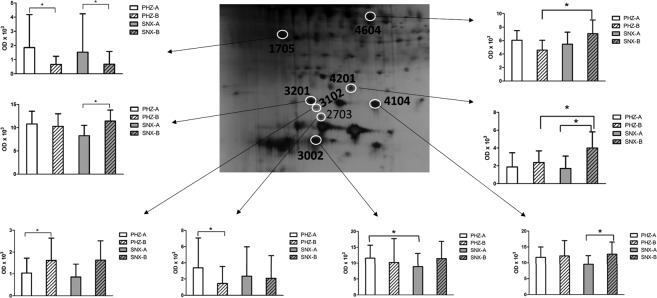
Isoforms of haptoglobin and haptoglobin-related protein. Quantitative data for the different haptoglobin and haptoglobin-related protein isoforms with molecular weight less than 50 kDa, detected in the interstitial fluid from human brain. Statistically significant changes (p < 0.05) indicated by*. The diagrams show the optical density (OD) as mean value (SD) for the different isoforms. To view the entire original image see Supplementary Fig. S8.

Several of the altered proteins identified by 2-DE/MS based analysis were identified as different isoforms of the same protein. For example, six different isoforms of haptoglobin, and two of haptoglobin-related protein were identified as being either up- or downregulated at the two locations and time points (Fig. 4).

#### Bioinformatics

**Figure 5:**
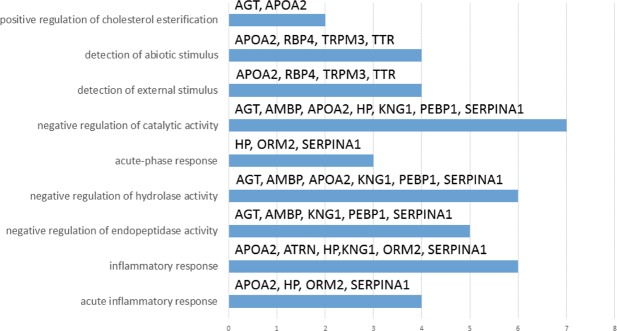
Pathway analysis of differentially expressed proteins (n=30) in PHZ-B compared to SNX-B. Significant (p = 2.11e-15) pathway analysis of differentially expressed proteins (n = 30) in PHZ-B compared to SNX-B. X-axis indicates the numbers of proteins and the annotations on the bars refer to protein name (Tables 2–4). On the Y-axis, the biological processes with false discovery rate < 0.05 are shown. The highest significant pathway (the acute inflammatory process) is at the bottom and the less significant (positive regulation of cholesterol esterification) is at the top of the Figure.

Network construction to organize the altered proteins was performed in order to find a significant interaction map using String (Search Tool for the Retrieval of Interacting Genes/proteins). The result indicated that the altered proteins were at least partially biologically connected, as a group (p = 2.11e-15; Fig. 5). The highest significant pathways that the altered proteins were involved in were inflammatory response (10 proteins), regulation of endopeptidase activity (5 proteins) and regulation of hydrolase activity (6 proteins).

## Discussion

The present report is the first to use proteomic analysis of interstitial fluid from patients surgically treated for intracerebral haemorrhage (ICH). Using microdialysis (MD), we were able to show that there are significant differences in protein expression depending on the distance from the MD catheter to the site of the evacuated hematoma and that these protein alterations evolve over time. Both gel-based and non-gel-based techniques together enabled the analysis of numerous proteins that were altered in ICH.

One strength of our study is the paired MD catheter design. The results obtained using a combination of an MD catheter in the vicinity of the ICH (the PHZ catheter) and one catheter in seemingly normal cortex (the SNX catheter), relatively unaffected by the ICH, imply that the observed protein changes are more likely to be explained by ICH-related secondary injury cascades than by MD catheter insertion artefacts. We aimed at placing the MD catheters at predetermined locations at time of ICH surgery.

Another strength is the standardised time from ICH onset to the second analysis, time interval B, which corresponds with the clinically relevant time of 72 hours after ICH. Time interval A (median 2–10 hours) was the earliest possible time interval after catheter placement. Regardless of design, there are two potential causes of brain injury; either it is caused by the ICH or by the surgical approach, or both. Also, heterogeneity in time from ICH onset to initiation of MD sampling is inevitable in a clinical ICH cohort like the present one. In view of that the first sampling showed only minor changes between the PHZ and the SNX catheter, our data reflect that the injury caused by the ICH is the most important for inducing protein changes in the surrounding brain tissue. Since the time from sampling start was different between the patients at the later time point, the temporal profile of low-molecular metabolites and sampled proteins could be different. However, alterations in protein concentration tend to happen over longer time compared to low-molecular metabolites which vary more rapidly, thereby the variability in the temporal profile of the patients is likely to have only a minor impact on protein alterations.

Low-molecular weight analytes show a pattern of metabolic disturbance in the PHZ compared to the SNX, with a persistent lactate/pyruvate ratio (LPR) elevation and lower glucose levels that did not reach the levels typically associated with ischemia^15^. These energy metabolic changes could contribute to an ongoing secondary injury process ultimately exacerbating the initial injury.

Our patient cohort consisted of nine male patients and one female patient. Thus, this cohort is too small to establish any gender-specific differences in the response to ICH. Gender differences in brain structure, function and chemistry have previously been described^33–35^ and in a previous traumatic brain injury (TBI) study the levels of glutamate and the lactate/pyruvate ratio in CSF were lower in females compared to males patients^36^. Similarly, significant gender differences on CSF markers of excitotoxicity, ischemia and oxidative damage were observed after TBI^37^. Potential gender differences in response to ICH should be evaluated in future studies.

Although there are no previous studies on the proteome of human brain following ICH, a study in a pig model of ICH showed differences in proteomic expression of whole tissue at 24 h and at 60 days compared to controls^38^. Another study in a rat model of acute ICH showed differential protein expression^39^, as did a tissue proteomic study of collagenase-induced ICH in rats at 2 days post-ICH^40^. These animal studies used whole tissue samples, and although interesting they present significant methodological differences to our present study which uses microdialysis for sampling, thus making direct comparisons difficult.

In total we were able to identify 30 proteins whose expression levels were changed in PHZ-B compared to SNX-B. The expression level of 18 proteins were decreased in PHZ-B and 12 proteins were increased compared to SNX-B. Bioinformatic analysis showed that the highest significant pathway that the altered proteins were involved in was an acute inflammatory process (Fig. 5). An inflammatory response and acute phase response following ICH has been previously described in patients as well as *in vivo* and *in vitro* studies^41–49^. The protein pathways identified in our present study give important insight into mechanisms of secondary brain injury present after ICH that could potentially be modified as a future strategy for the treatment of ICH.

Two such endogenous potentially neuroprotective proteins shown in our study to be significantly increased over time in the perihaemorrhagic zone are transthyretin and haptoglobin. Haptoglobin is involved with the scavenging of heme, and may have a role in ICH clearance, and has shown neuroprotective effects in animal models of ICH^50–52^. The levels of transthyretin, involved in the transport of vitamin A and thyroid hormones, was increased both in the SNX over time, but also in the PHZ compared to the SNX at time interval B in our study. This suggests that transthyretin is involved in an active process in the parenchyma adjacent to the ICH as well as globally in the brain tissue. This is in line with previous studies in human clinical studies and *in vivo* and *in vitro* studies suggesting a neuroprotective effect of transthyretin in Alzheimer’s disease and brain ischemia^53–55^, as well as a neurotrophic effect on neuronal regeneration^56^.

The present study is the first to combine a dual microdialysis catheter set-up with protein analysis of microdialysate from the human brain. A previous study using proteomic analysis (LC-based) of the plasma proteins of 8 ICH patients showed that 31 proteins were expressed in different abundance when compared to controls^57^, but only one of these proteins was significantly altered in our study (haptoglobin-related protein). This suggests that the expression of proteins differs distinctly between blood and the interstitial fluid of the brain. Another study used proteomic analysis of pooled MD samples from five patients suffering from traumatic brain injury and found an altered protein expression in patients with energy metabolic disturbance when compared to those without^58^. These altered proteins consisted of cytoarchitectural proteins, blood breakdown proteins as well as some mitochondrial proteins although no temporal resolution was provided. In a clinical TBI study, the proteome of cortical tissue biopsies differed between injury types^59^.

There are challenges when using MD for monitoring proteins, such as low abundance, low relative recovery and “sticky” proteins adhering to the MD membrane or tubing^60^. The Relative Recovery (RR), also termed Extraction Efficiency (EE), is crucial to consider in any MD study. The RR (%) is defined as the ratio of the concentration of a certain compound in the microdialysate to that of the tissue sampled^13^. Some proteins are present in very low abundance in the extracellular fluid making their recovery challenging. It is also possible that the RR changes over time, for example as the membrane gets saturated by proteins adhering to it. Since we used a study design with paired catheters and evaluated protein changes at two time points, it is plausible that the RR is similar between membranes. In addition the RR may be influenced by many factors such as membrane properties, the selected perfusate and the surrounding tissue pressure. Alterations of these factors may in unfavourable conditions drive the membrane’s properties towards a convective ultrafiltration flow and away from the diffusive flow across the membrane preferable for extracellular fluid sampling. The adherence of proteins to the membrane and the MD tubing may lead to a poor relative recovery and inability to detect proteins not eluted into the dialysate. To overcome this problem, the relative difference between samples was used instead of the absolute values in the statistical analysis to establish trends and patterns.

One limitation of the present study is the relatively small number of patients. We have identified alteration of several substances but it is too early to define these substances as biomarkers as the number of patients is small and the present methodological approach can only be used in a small subset of ICH patients. Further studies analysing these findings using e.g. ELISA, in a larger patient cohort are warranted.

Due to the use of dual MD catheters and two time-points, we were able to get robust statistical results. Patient characteristics were also relatively homogenous in this ICH cohort. Thus, the results of the present study can, despite the relatively small number of patients, therefore be considered valid and hypothesis generating for future research.

One limitation is that the catheters were not placed prior to surgery, which would be ethically indefensible, and thus we do not have any measurements of the energy metabolic state or protein expression prior to surgery. As a consequence, we cannot distinguish which proteomic changes were due to the surgical trauma and which ones were due to the ICH itself. Presumably, both the surgically induced trauma and the brain injury caused by the ICH contributed to the observed results.

Although a number of protein changes were detected, we performed the analysis on a selected number of proteins. It is therefore possible that more yet unidentified proteins are changed following ICH. As previously mentioned, a challenge in microdialysis is protein adsorption to the catheter membrane that may lead to the loss of important proteins. Future studies analysing both dialysate samples and proteins adsorbed to the catheter membrane might give improved protein coverage to identify potential biomarkers in ICH.

Importantly, there were large differences between the seemingly normal brain and in the perihaemorrhagic zone and some protein markers showed dynamic changes over time following onset of ICH. Our findings imply an ongoing pathophysiological process in the vicinity of an ICH which may contribute to an ongoing tissue injury.

## Conclusion

We used a novel approach and implanted dual microdialysis catheters, one in the vicinity of a surgically evacuated intracerebral haemorrhage (ICH) and one in a non-eloquent cortical area remote from the ICH. By applying proteomic analysis to the microdialysate, we were able to show a dynamic expression pattern of proteins presumably involved in the secondary injury cascades following ICH. These findings could aid in the understanding of secondary brain injury processes and in the elucidation of potential ICH biomarkers. Additional studies are needed in order to verify the findings of this study, and their potential use in the development of novel therapeutic targets.

## Supplementary information

Supplementary information

Dataset S3

## References

[1] Hemphill JC (2015). Guidelines for the Management of Spontaneous Intracerebral Hemorrhage: A Guideline for Healthcare Professionals From the American Heart Association/American Stroke Association. Stroke; a journal of cerebral circulation.

[2] Steiner T (2014). European Stroke Organisation (ESO) guidelines for the management of spontaneous intracerebral hemorrhage. International journal of stroke: official journal of the International Stroke Society.

[3] Mendelow AD (2013). Early surgery versus initial conservative treatment in patients with spontaneous supratentorial lobar intracerebral haematomas (STICH II): a randomised trial. Lancet.

[4] Prasad, K., Mendelow, A. D. & Gregson, B. Surgery for primary supratentorial intracerebral haemorrhage. *The Cochrane database of systematic reviews*, Cd000200, 10.1002/14651858.CD000200.pub2 (2008). 10.1002/14651858.CD000200.pub2 18843607

[5] Mendelow AD (2005). Early surgery versus initial conservative treatment in patients with spontaneous supratentorial intracerebral haematomas in the International Surgical Trial in Intracerebral Haemorrhage (STICH): a randomised trial. Lancet.

[6] Qureshi AI (2010). Effect of systolic blood pressure reduction on hematoma expansion, perihematomal edema, and 3-month outcome among patients with intracerebral hemorrhage: results from the antihypertensive treatment of acute cerebral hemorrhage study. Archives of neurology.

[7] Qureshi AI, Hanel RA, Kirmani JF, Yahia AM, Hopkins LN (2002). Cerebral blood flow changes associated with intracerebral hemorrhage. Neurosurgery clinics of North America.

[8] Qureshi AI, Wilson DA, Hanley DF, Traystman RJ (1999). No evidence for an ischemic penumbra in massive experimental intracerebral hemorrhage. Neurology.

[9] Kim-Han JS, Kopp SJ, Dugan LL, Diringer MN (2006). Perihematomal mitochondrial dysfunction after intracerebral hemorrhage. Stroke; a journal of cerebral circulation.

[10] Nilsson, O. G., Polito, A., Saveland, H., Ungerstedt, U. & Nordstrom, C. H. Are primary supratentorial intracerebral hemorrhages surrounded by a biochemical penumbra? A microdialysis study. *Neurosurgery* **59**, 521–528; discussion 521–528, 10.1227/01.neu.0000227521.58701.e5 (2006). 10.1227/01.NEU.0000227521.58701.E5 16955033

[11] Qureshi AI, Mendelow AD, Hanley DF (2009). Intracerebral haemorrhage. Lancet.

[12] Hillered L, Persson L, Nilsson P, Ronne-Engstrom E, Enblad P (2006). Continuous monitoring of cerebral metabolism in traumatic brain injury: a focus on cerebral microdialysis. Current opinion in critical care.

[13] Ungerstedt U (1991). Microdialysis–principles and applications for studies in animals and man. Journal of internal medicine.

[14] Diedler J (2010). Autoregulation and brain metabolism in the perihematomal region of spontaneous intracerebral hemorrhage: an observational pilot study. Journal of the neurological sciences.

[15] Tobieson, L., Rossitti, S., Zsigmond, P., Hillman, J. & Marklund, N. Persistent Metabolic Disturbance in the Perihemorrhagic Zone Despite a Normalized Cerebral Blood Flow Following Surgery for Intracerebral Hemorrhage. *Neurosurgery*, 10.1093/neuros/nyy179 (2018). 10.1093/neuros/nyy179 PMC6520101 29788388

[16] Hillman, J. *et al*. A microdialysis technique for routine measurement of macromolecules in the injured human brain. *Neurosurgery* **56**, 1264–1268; discussion 1268–1270 (2005). 10.1227/01.neu.0000159711.93592.8d 15918942

[17] Engstrom M (2005). Intracerebral microdialysis in severe brain trauma: the importance of catheter location. Journal of neurosurgery.

[18] Hutchinson P, O’Phelan K (2014). International multidisciplinary consensus conference on multimodality monitoring: cerebral metabolism. Neurocritical care.

[19] van Swieten JC, Koudstaal PJ, Visser MC, Schouten HJ, van Gijn J (1988). Interobserver agreement for the assessment of handicap in stroke patients. Stroke; a journal of cerebral circulation.

[20] Quinn TJ, Dawson J, Walters MR, Lees KR (2009). Reliability of the modified Rankin Scale: a systematic review. Stroke; a journal of cerebral circulation.

[21] Hutchinson PJ (2015). Consensus statement from the 2014 International Microdialysis Forum. Intensive care medicine.

[22] Hillman, J. *et al*. Intracerebral microdialysis in neurosurgical intensive care patients utilising catheters with different molecular cut-off (20 and 100 kD). *Acta neurochirurgica* **148**, 319–324; discussion 324, 10.1007/s00701-005-0670-8 (2006). 10.1007/s00701-005-0670-8 16411015

[23] Hutchinson PJ (2005). Cerebral microdialysis methodology–evaluation of 20 kDa and 100 kDa catheters. Physiological measurement.

[24] Bradford MM (1976). A rapid and sensitive method for the quantitation of microgram quantities of protein utilizing the principle of protein-dye binding. Analytical biochemistry.

[25] Gorg A (2000). The current state of two-dimensional electrophoresis with immobilized pH gradients. Electrophoresis.

[26] Shevchenko A, Wilm M, Vorm O, Mann M (1996). Mass spectrometric sequencing of proteins silver-stained polyacrylamide gels. Analytical chemistry.

[27] Olausson P, Gerdle B, Ghafouri N, Larsson B, Ghafouri B (2012). Identification of proteins from interstitium of trapezius muscle in women with chronic myalgia using microdialysis in combination with proteomics. PloS one.

[28] Turkina MV, Ghafouri N, Gerdle B, Ghafouri B (2017). Evaluation of dynamic changes in interstitial fluid proteome following microdialysis probe insertion trauma in trapezius muscle of healthy women. Scientific reports.

[29] Zybailov B (2006). Statistical analysis of membrane proteome expression changes in Saccharomyces cerevisiae. Journal of proteome research.

[30] Olausson P, Ghafouri B, Backryd E, Gerdle B (2017). Clear differences in cerebrospinal fluid proteome between women with chronic widespread pain and healthy women - a multivariate explorative cross-sectional study. Journal of pain research.

[31] Wheelock AM, Wheelock CE (2013). Trials and tribulations of ‘omics data analysis: assessing quality of SIMCA-based multivariate models using examples from pulmonary medicine. Molecular bioSystems.

[32] Ronne-Engstrom E (2001). Intracerebral microdialysis in neurointensive care: the use of urea as an endogenous reference compound. Journal of neurosurgery.

[33] Cosgrove KP, Mazure CM, Staley JK (2007). Evolving knowledge of sex differences in brain structure, function, and chemistry. Biological psychiatry.

[34] Birzniece V (2006). Neuroactive steroid effects on cognitive functions with a focus on the serotonin and GABA systems. Brain research reviews.

[35] McEwen BS (2001). Invited review: Estrogens effects on the brain: multiple sites and molecular mechanisms. Journal of applied physiology.

[36] Wagner AK (2005). Gender associations with cerebrospinal fluid glutamate and lactate/pyruvate levels after severe traumatic brain injury. Critical care medicine.

[37] Wagner AK (2004). Relationships between cerebrospinal fluid markers of excitotoxicity, ischemia, and oxidative damage after severe TBI: the impact of gender, age, and hypothermia. Journal of neurotrauma.

[38] Sidyakin AA, Kaysheva AL, Kopylov AT, Lobanov AV, Morozov SG (2018). Proteomic Analysis of Cerebral Cortex Extracts from Sus scrofa with Induced Hemorrhagic Stroke. Journal of molecular neuroscience: MN.

[39] Deng, S., Feng, S., Wang, W., Zhao, F. & Gong, Y. Biomarker and Drug Target Discovery Using Quantitative Proteomics Post-Intracerebral Hemorrhage Stroke in the Rat Brain. *Journal of molecular neuroscience: MN*, 10.1007/s12031-018-1206-z (2018). 10.1007/s12031-018-1206-z PMC6267379 30430305

[40] Liu T (2018). Quantitative proteomic analysis of intracerebral hemorrhage in rats with a focus on brain energy metabolism. Brain and behavior.

[41] Honig A, Leker RR (2018). Between a rock and hard place: fever and inflammation in intracerebral hemorrhage. European journal of neurology: the official journal of the European Federation of Neurological Societies.

[42] Lei C (2013). High-mobility group box 1 protein promotes neuroinflammation after intracerebral hemorrhage in rats. Neuroscience.

[43] Liu B (2015). CD163/Hemoglobin Oxygenase-1 Pathway Regulates Inflammation in Hematoma Surrounding Tissues after Intracerebral Hemorrhage. Journal of stroke and cerebrovascular diseases: the official journal of National Stroke Association.

[44] Mracsko E, Veltkamp R (2014). Neuroinflammation after intracerebral hemorrhage. Frontiers in cellular neuroscience.

[45] Wang J (2010). Preclinical and clinical research on inflammation after intracerebral hemorrhage. Progress in neurobiology.

[46] Wang J, Dore S (2007). Inflammation after intracerebral hemorrhage. Journal of cerebral blood flow and metabolism: official journal of the International Society of Cerebral Blood Flow and Metabolism.

[47] Zhou Y, Wang Y, Wang J, Anne Stetler R, Yang QW (2014). Inflammation in intracerebral hemorrhage: from mechanisms to clinical translation. Progress in neurobiology.

[48] Yang Y (2016). Attenuation of Acute Phase Injury in Rat Intracranial Hemorrhage by Cerebrolysin that Inhibits Brain Edema and Inflammatory Response. Neurochemical research.

[49] Di Napoli M (2018). Monomeric C-Reactive Protein and Cerebral Hemorrhage: From Bench to Bedside. Frontiers in immunology.

[50] Zhao X (2009). Neuroprotective role of haptoglobin after intracerebral hemorrhage. The Journal of neuroscience: the official journal of the Society for Neuroscience.

[51] Zhao X (2011). Cytoprotective role of haptoglobin in brain after experimental intracerebral hemorrhage. Acta neurochirurgica. Supplement.

[52] Wang G (2018). PPAR-gamma Promotes Hematoma Clearance through Haptoglobin-Hemoglobin-CD163 in a Rat Model of Intracerebral Hemorrhage. Behavioural neurology.

[53] Silva CS (2017). Transthyretin neuroprotection in Alzheimer’s disease is dependent on proteolysis. Neurobiology of aging.

[54] Buxbaum J, Koziol J, Connors LH (2008). Serum transthyretin levels in senile systemic amyloidosis: effects of age, gender and ethnicity. Amyloid.

[55] Santos SD (2010). CSF transthyretin neuroprotection in a mouse model of brain ischemia. Journal of neurochemistry.

[56] Gomes JR (2016). Transthyretin provides trophic support via megalin by promoting neurite outgrowth and neuroprotection in cerebral ischemia. Cell death and differentiation.

[57] Li GC (2017). Identification of novel biomarker and therapeutic target candidates for acute intracerebral hemorrhage by quantitative plasma proteomics. Clinical proteomics.

[58] Lakshmanan R (2010). Metabolic crisis after traumatic brain injury is associated with a novel microdialysis proteome. Neurocritical care.

[59] Abu Hamdeh S (2018). Proteomic differences between focal and diffuse traumatic brain injury in human brain tissue. Scientific reports.

[60] Dahlin AP (2010). Methodological aspects on microdialysis protein sampling and quantification in biological fluids: an *in vitro* study on human ventricular CSF. Analytical chemistry.

