# Supplementary material for: Dynamic protein changes in the perihaemorrhagic zone of Surgically Treated Intracerebral Haemorrhage Patients
Source: Sci Rep. 2019 Feb 28;9:3181. doi: 10.1038/s41598-019-39499-2 (PMC6395593; doi:10.1038/s41598-019-39499-2)
Supplement: Supplementary file 1 — Supplementary information [file 41598_2019_39499_MOESM1_ESM.docx]

# Dynamic protein changes in the perihaemorrhagic zone of surgically treated intracerebral haemorrhage patients

Lovisa Tobieson^1*^, Bijar Ghafouri^2^, Peter Zsigmond^1^, Sandro Rossitti^1^, Jan Hillman^1^ Niklas Marklund^1,3^

^1^ Department of Neurosurgery and Department of Clinical and Experimental Medicine, Linköping University, Linköping, Sweden.

^2^ Division of Community Medicine, Department of Medical and Health Sciences, Linköping University, Pain and Rehabilitation Center, Anaesthetics, Operations and Specialty Surgery Centre, Region Östergötland, Linköping, SE-58185 Sweden

^3^ Lund University, Skåne University Hospital, Department of Clinical Sciences Lund, Neurosurgery, Lund, SE-22185 Sweden

*Correspondence to lovisa.tobieson@regionostergotland.se

SUPPLEMENTARY MATERIAL:

Supplementary Table S1. Table. Vial numbers pulled for each analysis method for every patient.

Supplementary Figure S2, Figure: Low-molecular weight metabolites lactate (A), pyruvate (B), glycerol (C), glutamate (D) and urea (E) for the first 84 hours of microdialysis sampling.

Supplementary Dataset S3, Data: Report of identified proteins in microdialysate samples from the human perihaemorrhagic brain using LC-MS/MS and Scaffold software.

Supplementary Figure S4, Figure. Pie chart classifying the identified proteins according to their biological processes. The identified proteins were grouped according to GO annotations.

Supplementary Table S5A and S5B. Table. Table A. Significant proteins in PHZ-B compared to SNX-B identified by 2DE/MS based proteomics. The optical density (OD) for the protein spots are presented as mean (standard deviation). Table B. Significant protein in PHZ-B compared to SNX-B identified by LC-MS based proteomics. The peak intensity for each protein is presented as mean (standard deviation).

Supplementary Figure S6A and S6B, Figure. The distribution profile for the 12 most important proteins (VIP>1) discriminating between PHZ-B and SNX-B. The bars represent the quantified expression level of each protein in the ten different individual samples.

Supplementary Table S7, Table. Known or assumed biological actions and processes of proteins in table 2 and 3. (Adapted from STRING Consortium data base).

Supplementary Figure S8, Figure. Original image of the 2D-PAGE gel which is shown and annotated in Figure 2 and Figure 4 in the main text.

Supplementary Table S1.

| Patient number | Time point | Vial # Gel-based | Vial # LC-MS/MS |
| --- | --- | --- | --- |
| 1 | A | 1+2 | 3+4 |
|  | B | 29+30 | 28+31 |
| 2 | A | 1+2 | 3+4 |
|  | B | 22+23 | 21+24 |
| 3 | A | 1+2 | 3+4 |
|  | B | 32+33 | 31+34 |
| 4 | A | 1+2 | 4+6 |
|  | B | 22+23 | 18+20 |
| 5 | A | 1+2 | 4+6 |
|  | B | 10+11 | 8+12+14 |
| 6 | A | 1+2 | 4+5 |
|  | B | 16+17 | 15+18 |
| 7 | A | 1+2 | 3+4 |
|  | B | 28+29 | 27+30 |
| 8 | A | 1+2 | 3+4 |
|  | B | 22+23 | 21+24 |
| 9 | A | 2+3 | 4+5 |
|  | B | 33+34 | 32+35 |
| 10 | A | 1+2 | 4+5 |
|  | B | 31+32 | 30+33 |

Table S1. Vial numbers pulled for each analysis method for each patient.

Supplementary Figure S2. Figure:


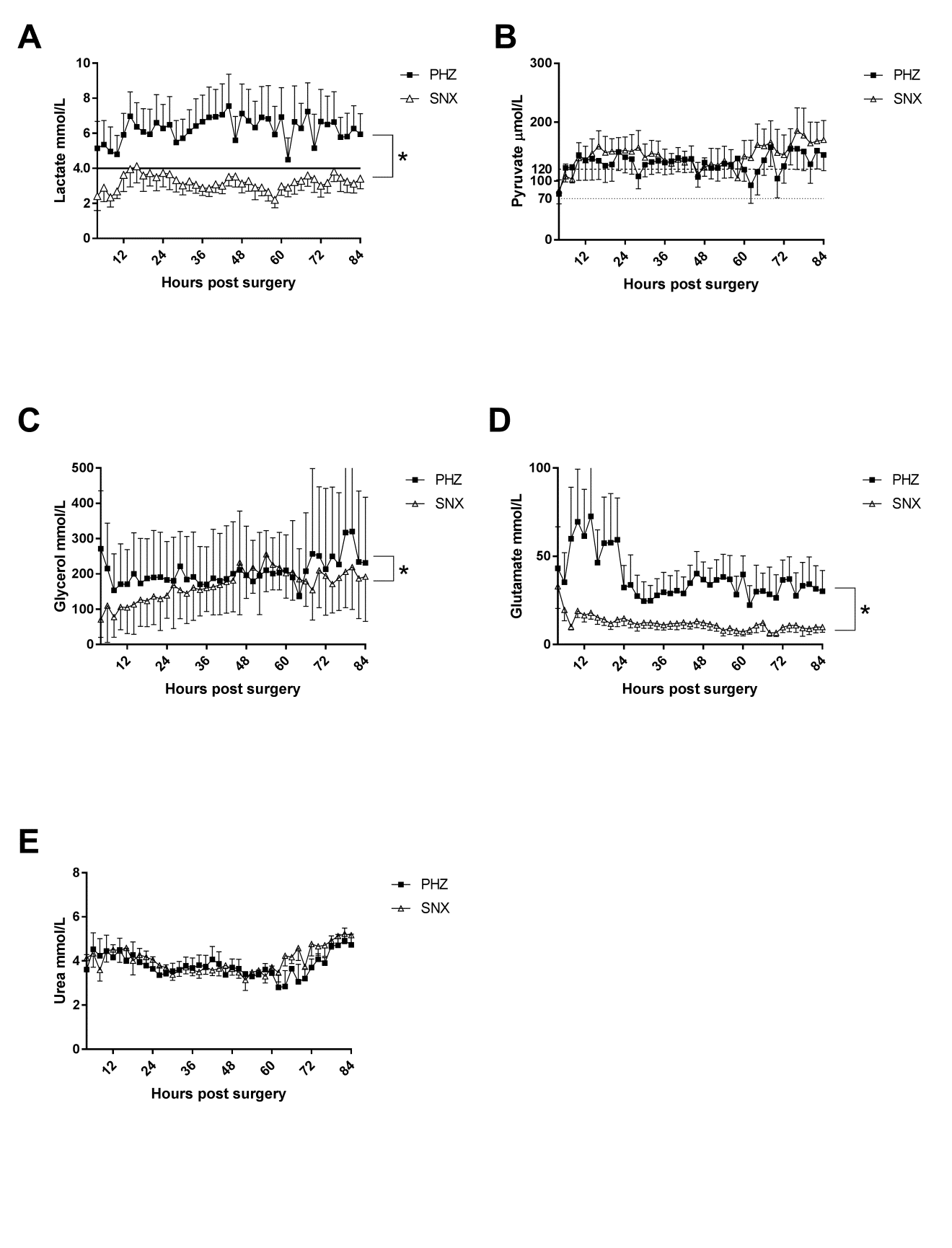


Low-molecular weight metabolites lactate (A), pyruvate (B), glycerol (C), glutamate (D) and urea (E) for the first 84 hours of microdialysis sampling. Error bars = standard error of the mean (S.E.M.). * indicates p<0.05.

Supplementary Figure S4. Figure.


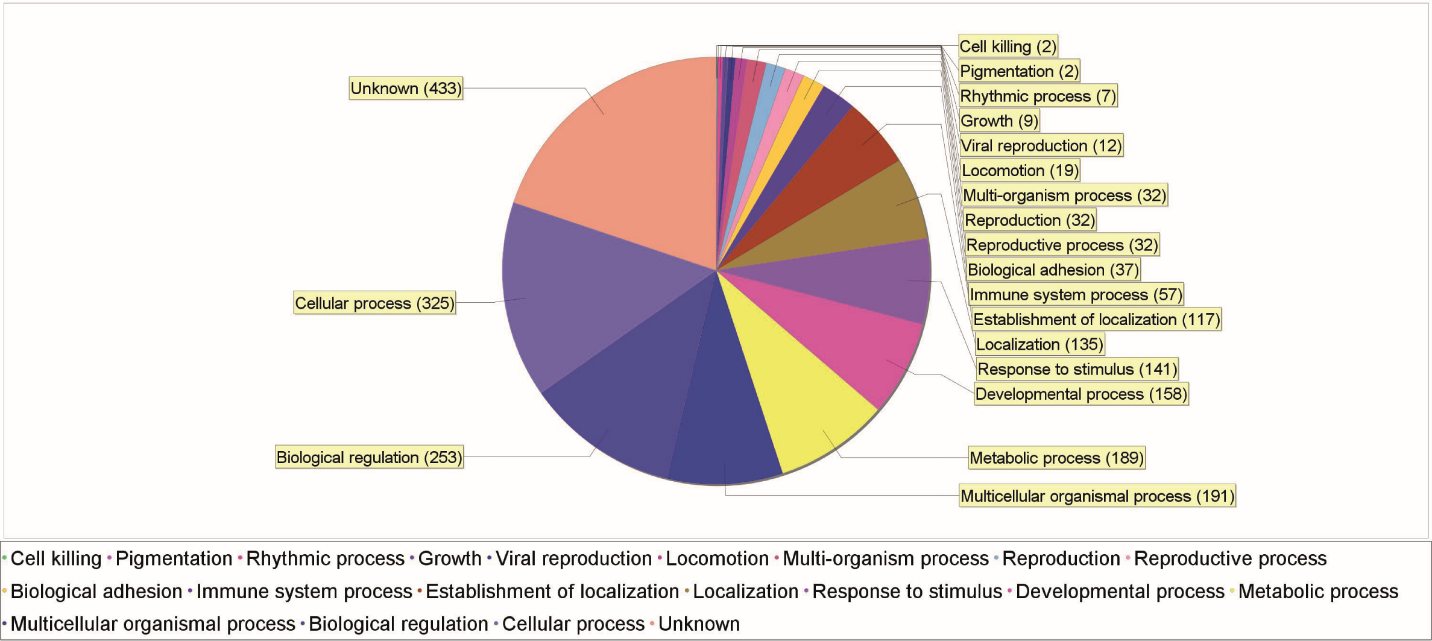
 Supplementary Figure S4. Figure: Pie chart classifying the identified proteins according to their biological processes. The identified proteins were grouped according to GO annotations.

Supplementary Table S5A

| Spot no. | UniProt ID | Protein namne | PHZ-B  OD mean (SD) | SNX-B  OD mean (SD) |
| --- | --- | --- | --- | --- |
| 4604 | P00738 | Haptoglobin | 4598 (482) | 7037 (638) |
| 6501 | Q96FN5 | Kinesin-like protein KIF12 | 2463 (434) | 3868 (329) |
| 5404 | Q6UXS9 | Inactive caspase-12 | 1841 (373) | 3686 (539) |
| 3119 | P02766 | Transthyretin | 123132(5923) | 101012 (17281) |
| 4105 | P02766 | Transthyretin | 206540 (13742) | 157213 (12395) |
| 4201 | P00739 | Haptoglobin-related protein | 2370 (435) | 3998 (570) |
| 7307 | P25189-2 | Myelin protein P0 | 1200 (227) | 2307 (423) |
| 3501 | P02760 | Protein AMBP | 6306 (1127) | 9357 (691) |
| 4205 | P01876 | Ig alpha-1 chain C region | 1709 (121) | 3289 (540) |
| 3205 | P15924 | Desmoplakin | 1749 (530) | 4070 (814) |
| 4203 | P01876 | Ig alpha-1 chain C region | 1110 (182) | 1705 (226) |
| 4407 |  | unidentified | 1297 (152) | 2489 (405) |

Table S5A. Significant proteins in PHZ-B compared to SNX-B identified by 2DE/MS based proteomics. The optical density (OD) for the protein spots are presented as mean (standard deviation).

Supplementary Table S5B

| Protein name | Uniprot | PHZ-B  Intensity mean (SD) | SNX-B  Intensity mean (SD) |
| --- | --- | --- | --- |
| Angiotensinogen | ANGT_HUMAN | 51651 (40942) | 13841 (28455) |
| Transthyretin | TTHY_HUMAN | 96123 (54540) | 196341 (114184) |
| Protocadherin Fat 4 | FAT4_HUMAN | 0.4 (0,5) | 0 (0) |
| Transient receptor potential cation channel subfamily M member 3 | TRPM3_HUMAN | 0.57 (0.57) | 0.11 (0.22) |
| Apolipoprotein A-II | APOA2_HUMAN | 85981 (43804) | 147251 (70928) |
| Kininogen-1 | KNG1_HUMAN | 41577 (23074) | 94636 (68486) |
| Alpha-1-antitrypsin | A1AT_HUMAN | 39989 (37224) | 16693 (16638) |
| Bromodomain and WD repeat-containing protein 1 | BRWD1_HUMAN | 0 (0) | 0.5 (0.6) |
| Fetuin-B | FETUB_HUMAN | 12547 (10673) | 4062 (7524) |
| Phosphatidylethanolamine-binding protein 1 | PEBP1_HUMAN | 10395 (16454) | 0 (0) |
| Protocadherin alpha-13 | PCDAD_HUMAN | 0.3 (0.5) | 0 (0) |
| Ig heavy chain V-III region CAM | HV307_HUMAN | 1614 (4270) | 0.6 (0.6) |
| Retinol-binding protein 4 | RET4_HUMAN | 88915 (61301) | 33410 (54956) |
| Ig kappa chain C region | IGKC_HUMAN | 17386 (18761) | 5500 (7787) |
| Hemopexin | HEMO_HUMAN | 138292 (65651) | 253412 (189073) |
| Ceruloplasmin | CERU_HUMAN | 68759 (30670) | 76214 (79388) |
| Alpha-1-acid glycoprotein 2 | A1AG2_HUMAN | 164886 (195748) | 362477 (303090) |
| Attractin | ATRN_HUMAN | 42174 (25864) | 50173 (78050) |
| Vitamin D-binding protein | VTDB_HUMAN | 23953 (28775) | 26000 (46084) |
| Ephrin type-A receptor 4 | EPHA4_HUMAN | 103662 (168127) | 5902 (16693) |

Table S5B. Significant protein in PHZ-B compared to SNX-B identified by LC-MS based proteomics. The peak intensity for each protein is presented as mean (standard deviation).

Supplementary figure S6A


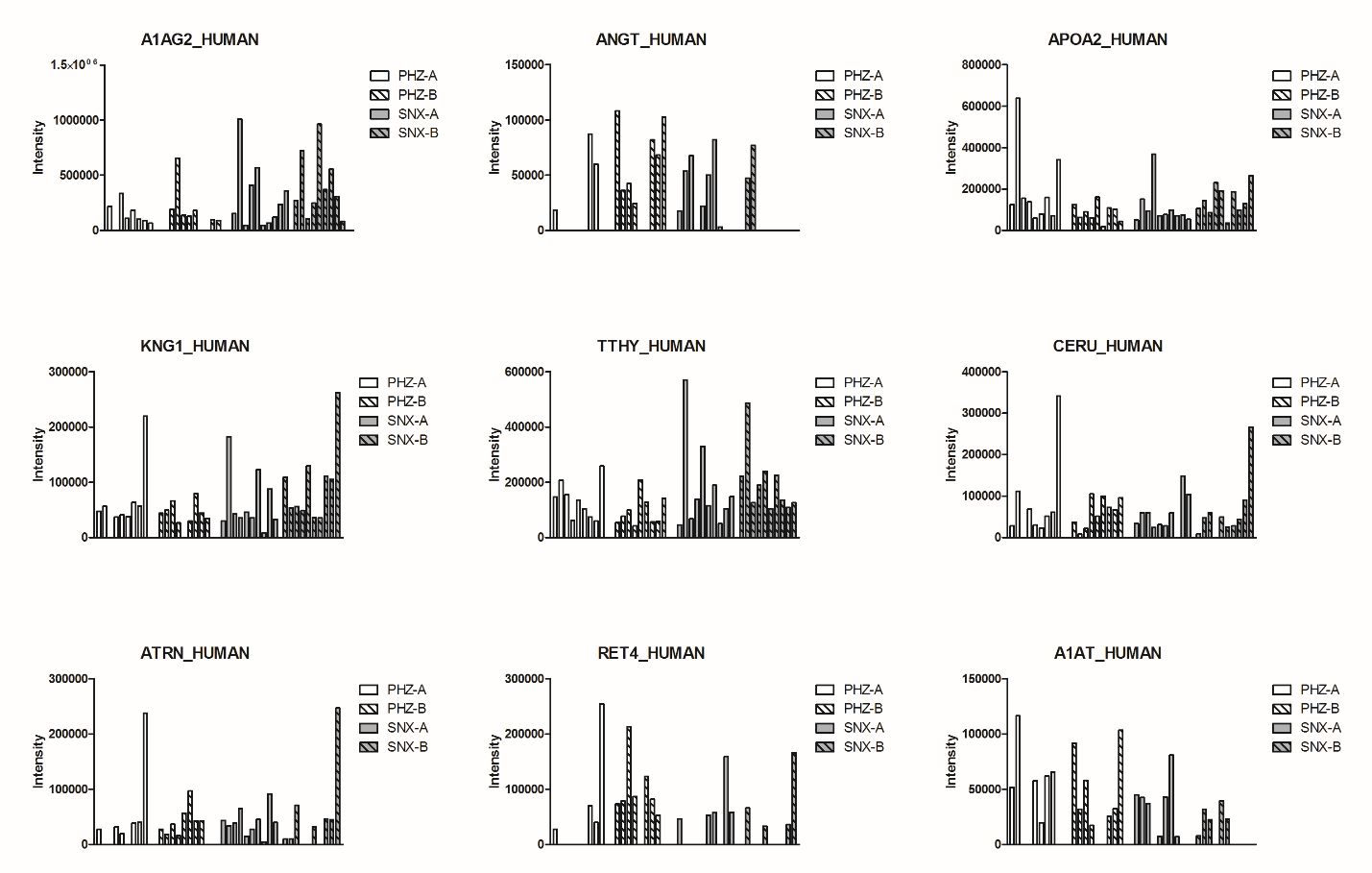


Supplementary figure S6A: The distribution profile for the 12 most important proteins (VIP>1) discriminating between PHZ-B and SNX-B. The bars represent the quantified expression level of each protein in the ten different individual samples.

Supplementary figure S6B.


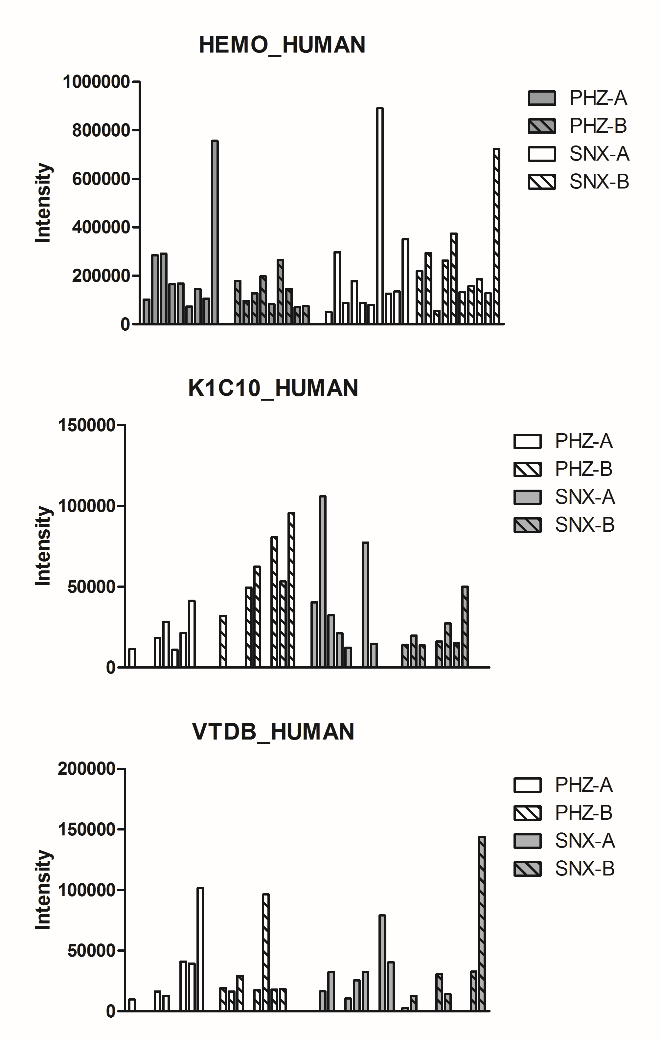


Supplementary figure S6B. Figure. The distribution profile for the 12 most important proteins (VIP>1) discriminating between PHZ-B and SNX-B. The bars represent the quantified expression level of each protein in the ten different individual samples.

Supplementary Table S7. Table. Known or assumed biological actions and processes of proteins in table 2 and 3 of the main text. (Adapted from STRING Consortium data base).

| APOH | Apolipoprotein H (beta-2-glycoprotein I); Binds to various kinds of negatively charged substances such as heparin, phospholipids, and dextran sulfate. May prevent activation of the intrinsic blood coagulation cascade by binding to phospholipids on the surface of damaged cells (345 aa) |
| --- | --- |
| TTR | Transthyretin; Thyroid hormone-binding protein. Probably transports thyroxine from the bloodstream to the brain (147 aa) |
| C3 | Complement component 3; C3 plays a central role in the activation of the complement system. Its processing by C3 convertase is the central reaction in both classical and alternative complement pathways. After activation C3b can bind covalently, via its reactive thioester, to cell surface carbohydrates or immune aggregates (1663 aa) |
| PHC1 | Polyhomeotic homolog 1 (Drosophila); Component of a Polycomb group (PcG) multiprotein PRC1- like complex, a complex class required to maintain the transcriptionally repressive state of many genes, including Hox genes, throughout development. PcG PRC1 complex acts via chromatin remodeling and modification of histones; it mediates monoubiquitination of histone H2A ’Lys-119’, rendering chromatin heritably changed in its expressibility (1004 aa) |
| PEBP1 | Phosphatidylethanolamine binding protein 1; Binds ATP, opioids and phosphatidylethanolamine. Has lower affinity for phosphatidylinositol and phosphatidylcholine. Serine protease inhibitor which inhibits thrombin, neuropsin and chymotrypsin but not trypsin, tissue type plasminogen activator and elastase (By similarity). Inhibits the kinase activity of RAF1 by inhibiting its activation and by dissociating the RAF1/MEK complex and acting as a competitive inhibitor of MEK phosphorylation (187 aa) |
| ITGB3 | Integrin, beta 3 (platelet glycoprotein IIIa, antigen CD61); Integrin alpha-V/beta-3 is a receptor for cytotactin, fibronectin, laminin, matrix metalloproteinase-2, osteopontin, osteomodulin, prothrombin, thrombospondin, vitronectin and von Willebrand factor. Integrin alpha-IIb/beta-3 is a receptor for fibronectin, fibrinogen, plasminogen, prothrombin, thrombospondin and vitronectin. Integrins alpha-IIb/beta-3 and alpha-V/beta-3 recognize the sequence R-G-D in a wide array of ligands. Integrin alpha-IIb/beta-3 recognizes the sequence H-H-L-G-G-G-A-K-Q-A-G-D-V in fibrinogen gamma chain. [...] (788 aa) |
| KNG1 | Kininogen 1; (1) Kininogens are inhibitors of thiol proteases; (2) HMW-kininogen plays an important role in blood coagulation by helping to position optimally prekallikrein and factor XI next to factor XII; (3) HMW-kininogen inhibits the thrombin- and plasmin- induced aggregation of thrombocytes; (4) the active peptide bradykinin that is released from HMW-kininogen shows a variety of physiological effects- (4A) influence in smooth muscle contraction, (4B) induction of hypotension, (4C) natriuresis and diuresis, (4D) decrease in blood glucose level, (4E) it is a mediator of inflammation [...] (644 aa) |
| FETUB | Fetuin B (382 aa) |
| AMBP | Alpha-1-microglobulin/bikunin precursor; Inter-alpha-trypsin inhibitor inhibits trypsin, plasmin, and lysosomal granulocytic elastase. Inhibits calcium oxalate crystallization (352 aa) |
| PCDHA13 | Protocadherin alpha 13; Potential calcium-dependent cell-adhesion protein. May be involved in the establishment and maintenance of specific neuronal connections in the brain (By similarity) (950 aa) |
| DCD | Dermcidin; DCD-1 displays antimicrobial activity thereby limiting skin infection by potential pathogens in the first few hours after bacterial colonization. Highly effective against E.coli, E.faecalis, S.aureus and C.albicans. Optimal pH and salt concentration resemble the conditions in sweat. Also exhibits proteolytic activity (110 aa) |
| ANKHD1 | Ankyrin repeat and KH domain containing 1 (2617 aa) |
| BRWD1 | Bromodomain and WD repeat domain containing 1 (2320 aa) |
| HBB | Hemoglobin, beta (147 aa) |
| ZFAND4 | Zinc finger, AN1-type domain 4 (727 aa) |
| SERPINA1 | Serpin peptidase inhibitor, clade A (alpha-1 antiproteinase, antitrypsin), member 1 (418 aa) |
| HP | Haptoglobin; As a result of hemolysis, hemoglobin is found to accumulate in the kidney and is secreted in the urine. Haptoglobin captures, and combines with free plasma hemoglobin to allow hepatic recycling of heme iron and to prevent kidney damage. Haptoglobin also acts as an Antimicrobial; Antioxidant, has antibacterial activity and plays a role in modulating many aspects of the acute phase response. Hemoglobin/haptoglobin complexes are rapidly cleared by the macrophage CD163 scavenger receptor expressed on the surface of liver Kupfer cells through an endocytic lysosomal degradation [...] (406 aa) |
| SON | SON DNA binding protein (2426 aa) |
| AGT | Angiotensinogen (serpin peptidase inhibitor, clade A, member 8); Essential component of the renin-angiotensin system (RAS), a potent regulator of blood pressure, body fluid and electrolyte homeostasis (485 aa) |
| DLL1 | Delta-like 1 (Drosophila); Acts as a ligand for Notch receptors. Blocks the differentiation of progenitor cells into the B-cell lineage while promoting the emergence of a population of cells with the characteristics of a T-cell/NK-cell precursor (723 aa) |
| APOA2 | Apolipoprotein A-II; May stabilize HDL (high density lipoprotein) structure by its association with lipids, and affect the HDL metabolism (100 aa) |
| RBP4 | Retinol binding protein 4, plasma; Delivers retinol from the liver stores to the peripheral tissues. In plasma, the RBP-retinol complex interacts with transthyretin, this prevents its loss by filtration through the kidney glomeruli (201 aa) |
| KIF12 | Kinesin family member 12 (513 aa) |
| TRPM3 | Transient receptor potential cation channel, subfamily M, member 3 (1707 aa) |
| DSP | Desmoplakin; Major high molecular weight protein of desmosomes. Involved in the organization of the desmosomal cadherin- plakoglobin complexes into discrete plasma membrane domains and in the anchoring of intermediate filaments to the desmosomes (2871 aa) |
| FAT4 | FAT tumor suppressor homolog 4 (Drosophila); May function in the regulation of planar cell polarity (By similarity). Cadherins are cell-cell interaction molecules (By similarity) (4981 aa) |
| CRISP3 | Cysteine-rich secretory protein 3 (268 aa) |
| KLHL5 | Kelch-like 5 (Drosophila) (755 aa) |
| CASP12 | Caspase 12 (gene/pseudogene); Has no protease activity. May reduce cytokine release in response to bacterial lipopolysaccharide during infections. Reduces activation of NF-kappa-B in response to TNF (341 aa) |
| MPZ | Myelin protein zero; Creation of an extracellular membrane face which guides the wrapping process and ultimately compacts adjacent lamellae (248 aa) |
| MUC5B | Mucin 5B, oligomeric mucus/gel-forming (5762 aa) |
| HPR | Haptoglobin-related protein (348 aa) |

Supplementary Figure S8
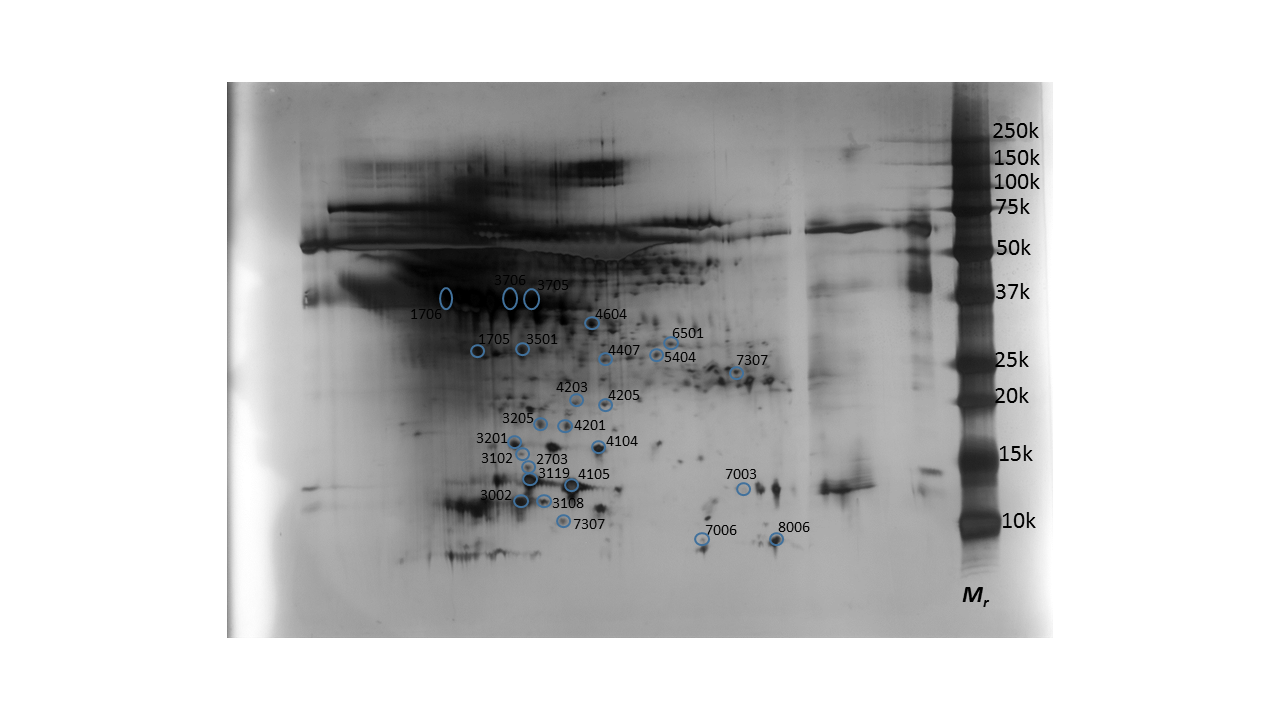


Supplementary Figure S8. Figure. Original image of the 2D-PAGE gel which is shown and annotated in Figure 2 and Figure 4 in the main text
